# The association between food desert severity, socioeconomic status, and metabolic state during pregnancy in a prospective longitudinal cohort

**DOI:** 10.1038/s41598-023-32783-2

**Published:** 2023-05-03

**Authors:** Elizabeth K. Wood, Gayle Stamos, A J Mitchell, Rose Gonoud, Angela M. Horgan, Olivia Nomura, Anna Young, Joel T. Nigg, Hanna C. Gustafsson, Elinor L. Sullivan

**Affiliations:** 1 Department of Psychiatry, Oregon Health & Science University, Portland, USA; 2 Department of Behavioral Neuroscience, Oregon Health & Science University, Portland, USA; 3 Clinical & Translational Research Center, Oregon Health & Science University, Portland, USA; 4 Division of Neuroscience, Oregon National Primate Research Center, Portland, USA

**Keywords:** Metabolic disorders, Diabetes, Obesity, Pre-diabetes

## Abstract

Poor metabolic health during pregnancy is associated with health concerns for pregnant individuals and their offspring. Lower socioeconomic status (SES) is one risk factor for poor metabolic health, and may be related to limited access to healthful and affordable foods (e.g., living in a food desert). This study evaluates the respective contributions of SES and food desert severity on metabolic health during pregnancy. The food desert severity of 302 pregnant individuals was determined using the United States Department of Agriculture Food Access Research Atlas. SES was measured using total household income adjusted for household size, years of education, and amount of reserve savings. Information about participants’ glucose concentrations one hour following an oral glucose tolerance test during the second trimester was extracted from medical records and percent adiposity during the second trimester was assessed using air displacement plethysmography. Information about participants’ nutritional intake during the second trimester was obtained by trained nutritionists via three unannounced 24-h dietary recalls. Structural equation models showed that lower SES predicted higher food desert severity (β = − 0.20, *p* = 0.008) and higher adiposity (β = − 0.27, *p* = 0.016) and consumption of a more pro-inflammatory diet (β = − 0.25, *p* = 0.003) during the second trimester of pregnancy. Higher food desert severity also predicted higher percent adiposity during the second trimester (β = 0.17, *p* = 0.013). Food desert severity significantly mediated the relationship between lower SES and higher percent adiposity during the second trimester (β_indirect_ = − 0.03, 95% CI [− 0.079, − 0.004]). These findings indicate that access to healthful and affordable foods is a mechanism by which SES contributes to adiposity during pregnancy and may inform interventions intended to improve metabolic health during pregnancy.

## Introduction

### Metabolic health in pregnancy

Metabolic disorders during pregnancy are associated with serious long-term health complications to the pregnant individual, including cardiometabolic disorders^1,2^, renal disease^3^, and urological conditions^4^. They also predict a host of birth and long-term complications for offspring, including pre-term birth^5^, stillbirth and neonatal death^6^, altered birthweights (higher, in the case of gestational diabetes^7,8^ and lower, in the case of pre-eclampsia^9^), and macrosomia^10^, poor metabolic health at birth^11^, long-term obesity^12,13^, metabolic conditions^14,15^, and an increased risk for neurodevelopmental disorders^16,17^. Defining the determinants of metabolic health during pregnancy is critical to improving perinatal and long-term health for pregnant people and their children.

### The impact of nutrition and living in a food desert on metabolic health

Data from non-obstetric populations suggest that access to healthy and affordable foods is associated with metabolic health. Those living in low-income areas with limited access to healthful foods (termed “food deserts”) are more likely to consume foods with poorer nutritional quality, when compared to those living in higher-income, higher-access areas^18^. Studies show that living in a food desert is associated with risk for obesity^19^ and nutritional deficiencies^20^. Few studies have assessed the relationship between food deserts and metabolic health during pregnancy; however, one study showed that living in a food desert was associated with increased likelihood of morbidity during pregnancy^21^, including metabolic disorders and another suggested decreased risk for gestational diabetes mellitus (GDM) in areas with more grocery stores^22^.

Food desert-mediated differences in dietary quality may be responsible for these associations. Among pregnant populations, some studies show that consumption of a more pro-inflammatory diet is associated with increased risk for GDM^23,24^. However, another study observed that a more pro-inflammatory diet is associated with decreased risk for GDM^25^. This heterogeneity may be related to differences in dietary data collection methods, with one study using self-administered food frequency questionnaires (FFQs) in the United States^25^, while the others utilized FFQs completed with the assistance of trained dietitians/interviewers among people in Iran and China^23,24^. While living in a food desert likely informs dietary quality, whether the inflammatory quality of the prenatal diet is related to living in a food desert during pregnancy has not, to our knowledge, been previously examined.

### The influence of socioeconomic status on metabolic health in pregnancy

Lower socioeconomic status (SES), an important correlate of health inequalities^26^, is associated with poorer health outcomes for pregnant people. Compared to those with higher SES, pregnant people with lower SES show an increased risk for gestational diabetes^27^, gestational weight gain^28^, obesity^29^, and metabolic syndrome^30^. The mechanisms by which low SES influences metabolic health are complex and multi-faceted (see^31^). Lower SES is associated with both reduced access to affordable, healthy foods^32^ and more pro-inflammatory diets^33,34^, which have been linked to metabolic health in non-obstetric populations, including increased risk for obesity^35^, hyperglycemia^36^, and metabolic syndrome^37^. Low SES is also associated with inadequate prenatal care^38^ and chronic stress^39^, as well as insufficient access to specific nutritive foods^40^ or food altogether^41^. Whether living in a food desert mediates the association between SES and metabolic health for pregnant people has not yet been articulated.

### Socioeconomic status, food desert severity, and their interactive impact on metabolic health in pregnancy

Identifying contributing factors that help explain the relationship between SES and metabolic health during pregnancy is important for optimizing intervention strategies for pregnant people with lower SES, as well as for reducing the burden of poor metabolic health during pregnancy on offspring. Living in a food desert may be one mechanism underlying the relationship between variation in SES and metabolic health during pregnancy. We seek to extend studies showing that lower SES is associated with reduced access to affordable, healthy foods^18,32^ and with increased pregnancy morbidity^27–30,42^ by examining food desert severity as a potential mediator of the relationship between SES and metabolic health outcomes during pregnancy. For example, access to fewer financial resources may lead to reduced access to a healthful diet, which may, in turn, impact metabolic health by increasing adiposity, promoting peripheral inflammation, and/or disrupting glucose regulation.

### Current study

We propose that, in addition to being mechanistically linked, both SES and food desert severity may play a role in metabolic health and nutrition during pregnancy, an association that has not, to our knowledge, been formally tested. While many other studies of food deserts utilize a dichotomous assessment of food desert status (living in a food desert vs. not living in a food desert)^43^, the use of a categorical variable does not capture the degree to which healthful and affordable foods are accessible and may limit the ability to detect meaningful differences. Thus, in the current study, we calculate the degree of food desert severity for each participant relative to others in their neighborhoods, allowing us to draw inferences about pregnancy metabolic health according to the spectrum of resource accessibility. We examine three outcomes related to metabolic health and nutritional quality in pregnancy: adiposity, glucose regulation, and the inflammatory nature of the prenatal diet. As these outcomes are linked to one another^44^, and provide complimentary, but distinct information, unlike previous work, we control for their interdependence using structural equation modeling (SEM). Furthermore, as there is a graded, positive linear association between maternal hyperglycemia and risk for adverse perinatal outcomes^45^, we examine a continuous measure of glucose regulation. As both low SES and living in a food desert are associated with consumption of a less healthful diet^46,47^, we also examine the effects of low SES and food desert severity on nutrition during pregnancy.

While the relationship between food desert severity and SES is complex and likely interrelated, elucidating the interacting role of each of these is critical for both practitioners and policy-makers as we work together to address disparate access to resources among pregnant individuals. Our analytic strategy is key to disentangling the unique role of SES and food desert severity on the metabolic and nutrition variables, as SEM allows us to simultaneously estimate the effects of SES and food desert severity on the metabolic and nutrition variables of interest, while controlling for their interdependence. Thus, we are able to examine the significance and strength of the different relationships in the context of the complete model (one in which SES and food desert severity are both present). Our use of SEM also limits the risk of multicollinearity impacting our results, as SEM is known to be robust to multicollinearity^48^. SEM also allows us to reduce measurement error by explicitly modeling shared variance, thus more valid coefficients are obtained. Importantly, SEM also allows us to examine *how* SES may impact metabolic health and dietary intake during pregnancy by assessing the mediating and mechanistic role of food desert severity.

In the current study, we examined whether food desert severity mediates the relationship between SES and metabolic health during pregnancy. This study had three hypotheses:

1. SES will have a negative relationship with metabolic health during pregnancy, with lower SES associated with increased adiposity, higher glucose concentrations during a routine glucose tolerance test (an indication of poorer glucose regulation), and consumption of a more pro-inflammatory diet during pregnancy.
2. Food desert severity will have a positive relationship with adverse metabolic health, with higher food desert severity associated with increased adiposity, higher glucose concentrations in response to a routine glucose tolerance testing, and consumption of a more pro-inflammatory diet during pregnancy.
3. Food desert severity will mediate the relationship between SES and metabolic health and diet during pregnancy, such that lower SES will lead to a higher degree of food desert severity, which in turn, leads to worse metabolic health during pregnancy.

## Methods

### Procedure

Data came from an ongoing, prospective longitudinal study examining the influence of perinatal nutrition, adiposity, and metabolic state during pregnancy on offspring outcomes. Recruitment for the study began in 2018 and all pregnancies occurred between 2018 and 2021. Participant addresses and demographic information were self-reported at study enrollment and at 37 weeks’ gestation. At 22 ± 2 weeks gestation, participants completed questionnaires, reported on their dietary intake, and completed in-laboratory body composition assessments. Information about maternal health and metabolic state was extracted from medical records.

### Participants

Participants (*N* = 302), ages 18–40 (*M*_age_ = 31.94, SD = 4.23) were recruited through Oregon Health & Science University (OHSU), a major academic medical center located in Portland, Oregon. The majority of participants identified as non-Hispanic White (*n* = 221; 73.20%). For details about recruitment and exclusionary criteria, including a flow chart, see Supplementary Fig. S1.

### Measures

#### Glucose regulation

As part of their routine prenatal care, participants were screened for GDM via an oral glucose tolerance test (GTT). One-hour plasma glucose concentrations (mg/dL) were utilized in analyses as they are a common metric for predicting health outcomes^49^, including whether further GTT testing is necessary. As participants were assessed at varying times during pregnancy (8.14–32.57 weeks), the number of weeks’ gestation at the time of assessment was included as a covariate in all analyses that included GTT data. See Supplementary Materials for more information.

#### Adiposity

To measure body composition, at 22 weeks’ gestation (*M* = 22.53, SD = 1.41), air displacement plethysmography was utilized via the BOD POD^©^ Body Composition tracking system (Life Measurement, Inc.)^50,51^. As an excess of body fluid is typically observed during pregnancy^52^, estimates of fat and lean mass in the body were adjusted using previously-described equations^53^ and second trimester pregnancy-adjusted percent fat mass was utilized in analyses. See Supplementary Materials for more information.

#### Nutrition

Second trimester dietary intake was measured by three, non-consecutive 24-h diet recalls conducted by trained dietitians, using the multi-pass method^54^ and the Nutrition Data System for Research software (versions 2018–2020; University of Minnesota, Minneapolis, Minnesota). See Supplementary Materials for more information.

To capture the inflammatory nature of participants’ diets, Dietary Inflammatory Index (DII) scores, a metric of dietary quality that may be particularly relevant for metabolic health^55^, were calculated from the 24-h diet recalls. Positive values indicate a more pro-inflammatory diet^56^. See Supplementary Materials for more information.

#### Socioeconomic status

Self-reported information, including years of education completed, total annual combined household income adjusted for number of people in the household, and amount of savings after adjusting for debt was utilized to capture information about SES. See Supplementary Materials for more information.

#### Food desert severity score

Geographic information system mapping was utilized to identify food deserts. This method offers advantages over others (e.g., those that enumerate the number of healthy vs. unhealthy food vendors in the neighborhood) because it provides a more comprehensive characterization of community resources when determining if an individual lives in a food desert.

Participant addresses obtained at study enrollment were converted to latitude and longitude coordinates and mapped onto census tracts using data from the 2010 Census of the Population. Using the Food Access Research Atlas (FARA), published by the Economic Research Service of the United States Department of Agriculture (USDA^57^) and census data, low-income (at least 20% of the population of the census tract has a median family income at or below 80% of the metropolitan area or state median income) and low-access (at least 33% of the census tract or at least 500 individuals reside far from a supermarket) census tracts were identified. Detailed information about the sources of the data used to characterize census tracts as food deserts can be found online (https://www.ers.usda.gov/data-products/food-access-research-atlas/documentation/#data). Briefly, income data came from the 2014–2018 American Community Survey, urban or rural designation was from the 2019 urbanized area geographies, and two 2019 lists of stores (one including stores authorized to receive SNAP benefits and another list from Trade Dimensions TDLinx) were combined to produce a list of stores with affordable and nutritious food.

Approximately one-quarter of participants in this study met criteria for living in a food desert (*n* = 70, 23.18%), a higher percentage than the US population (12.8%)^58^. In order to capture the range of access to healthy and affordable foods, we calculated the degree of food desert severity for each participant relative to others in their census tract by dividing the number of individuals in a tract living more than 0.5 mile (urban areas) or 10 miles (rural areas) from the nearest supermarket, supercenter, or large grocery store) by the total number of individuals living within the tract. Food desert severity index was used as our primary analysis variable.

#### Covariates

As advanced age of birthing parent (defined by age at last menstrual period), increasing parity, and minoritized race or ethnicity are risk factors for poor metabolic health during pregnancy^59,60^, these variables were considered as covariates in analyses. Due to their relationships with SES and metabolic health^61–63^, we also examined alcohol use during pregnancy, as well as the use of prenatal vitamins. See Supplementary Materials for more information.

#### Analytic plan

Structural equation modeling (SEM) was conducted in M*plus* (v. 8; Muthen & Muthen, 1998–2021). SEM was selected because it allows us to simultaneously model the complex associations between multiple predictors and multiple outcomes, while controlling for their interdependence. Furthermore, this approach allows us to test for mediation. All data utilized in analyses were first screened by qualified experts, including a trained nutritionist, to assess normality and plausibility. With the exception of percent adiposity during the second trimester, nearly all variables of interest were non-normally distributed (Kolmogorov–Smirnov tests; *p*s < 0.05). Thus, a robust maximum likelihood estimator was utilized to accommodate for non-normal data distributions using a sandwich estimator. Mediation was tested using the model indirect command in M*plus*. As the use of conventional tests of significance are unreliable when testing indirect effects, we utilized the recommended asymmetric confidence intervals based on bootstrapping methods^64^ to incorporate the non-normality of the data into the model test statistics. Estimates and accompanying confidence intervals were calculated based on 10,000 bootstrapped samples. Missing data were handled using full information maximum likelihood^65^. Model fit was assessed by examining the comparative fit index (CFI; adequate fit was considered at CFI ≥ 0.90), the Tucker Lewis index (TLI; adequate fit was considered at TLI ≥ 0.90), the standardized root mean square residual (SRMR; adequate fit was considered at SRMR ≤ 0.05), and the root mean squared error of the approximation (RMSEA; adequate fit was considered at RMSEA ≤ 0.08)^66,67^. Non-independent observations (i.e., participants who were followed across multiple pregnancies; *n* = 5) were accounted for using the M*plus cluster* command. See Supplementary Materials for more information.

A stepwise approach to increasing model complexity was utilized:

1. *Data reduction* Prior to hypothesis testing, a confirmatory factor analysis was used to assess the appropriateness of considering our indicators of SES as a latent variable.
2. *Covariate selection* Covariates were examined and any that were significantly correlated (*p* < 0.05) with the indicators of SES, maternal metabolic variables, or food desert severity were included in the more complex models, as described below.
3. *Hypotheses 1 and 2: Regression Analyses.* To test Hypotheses 1 and 2, a series of regression models were run where: (a) food desert severity was regressed on SES; (b) pregnancy adiposity, glucose concentrations, and DII values were regressed on SES (each pregnancy metabolic variable was considered in its own model); and (c) pregnancy adiposity, glucose concentrations, and DII values were regressed on food desert severity (each pregnancy metabolic variable was considered in its own model).
4. *Hypothesis 3: Structural Equation Mediation Model.* To test whether food desert severity significantly mediated the relationship between SES and each of the pregnancy metabolic variables, an SEM was estimated in which each of the pregnancy metabolic variables were simultaneously regressed on SES and on food desert severity. In the same model, food desert severity was also regressed on SES. Pregnancy metabolic variables were allowed to covary, allowing for control of their interdependence. Each metabolic variable and food desert severity were regressed on each covariate and SES and each covariate were allowed to covary. To preserve model parsimony, non-significant paths from covariates were removed from the final model. Beta weights and *p*-values are reported for each of the paths, as well as bootstrapped confidence intervals for the indirect paths.

### Institutional review board statement

The study was conducted according to the guidelines of the Declaration of Helsinki, and approved by the Institutional Review Board of Oregon Health & Science University (IRB #18579; Date of approval: 06/05/2018).

### Informed consent statement

Informed consent was obtained from all subjects involved in the study.

## Results

### Sample description

**Figure 1:**
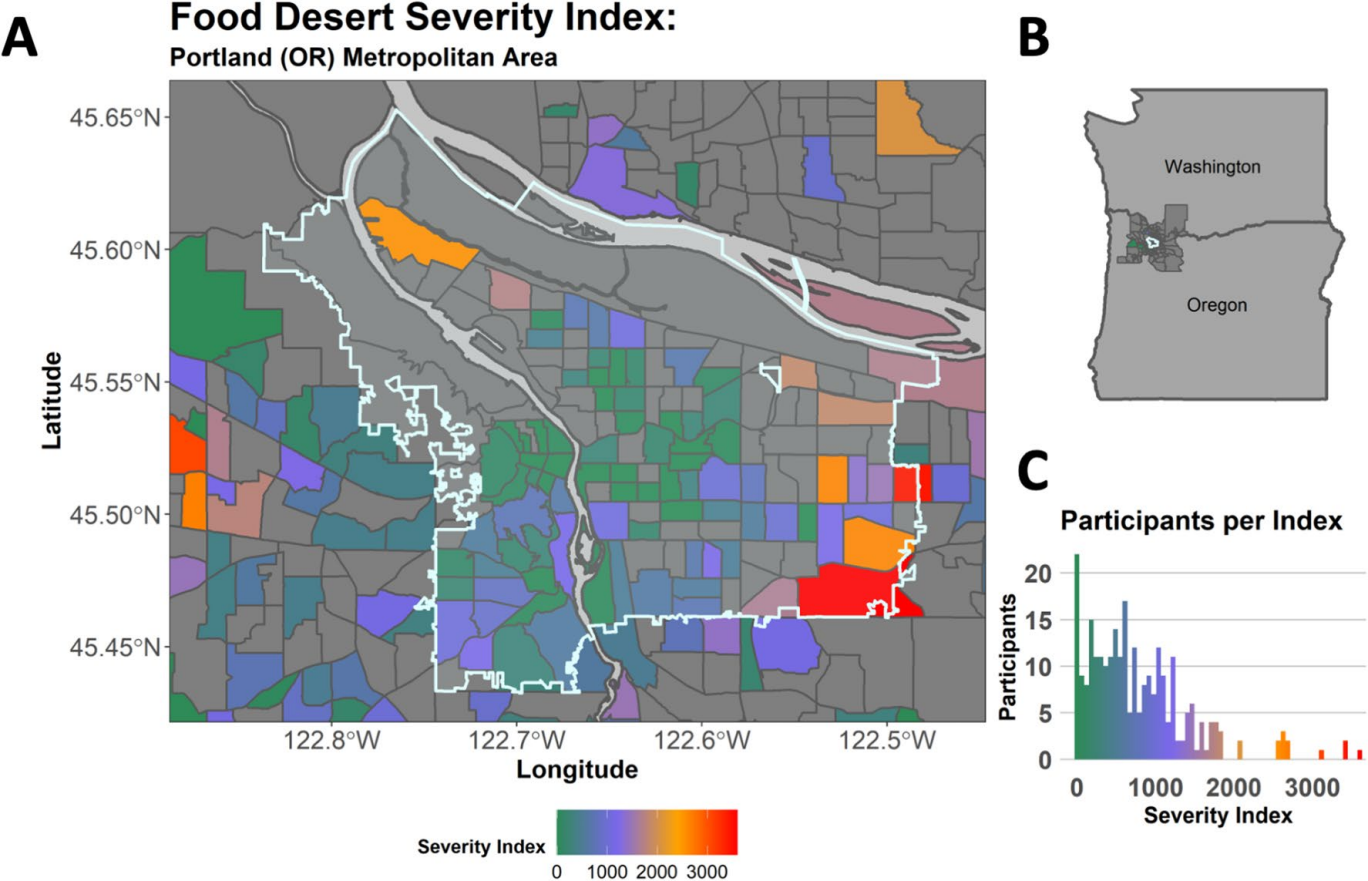
Food desert severity of participants in study. Figure displays food desert severity index for the participants in the study. Panel (**A**) displays a choropleth map representing the food desert severity index of census tracts within the metropolitan Portland, Oregon area. Panel (**B**) provides geographical orientation. Panel (**C**) displays a histogram of food desert severity index where values closer to zero indicate a lower food desert severity index. This figure was generated using R software (version 4.2.2; https://www.R-project.org/)^75^. The shape file was obtained from the City of Portland (https://gis-pdx.opendata.arcgis.com/) and based on 2010 Census boundaries. Integral R packages included tidyverse^76^, sf^77^, and ggmaps^78^.

Table 1 provides sample descriptive information, including means, standard deviations, and ranges for focal study variables. Figure 1 displays the food desert severity index of the different census tracts in which participant addresses at study enrollment were located.

**Table 1:** Descriptive statistics for study variables.

Variables	Total sample			Pregravid body composition^a^							
	*N*	Mean (SD) or %	Range or *n*	Underweight (*n* = 7)		Healthy weight (*n* = 181)		Overweight (*n* = 70)		Obese (*n* = 34)	
				Mean (SD) or %	Range	Mean (SD) or %	Range	Mean (SD) or %	Range	Mean (SD) or %	Range
Pregnancy adiposity (% body fat)	191	0.34 (0.07)	0.15–0.54	0.25 (0.06)	0.15–0.31	0.31 (0.04)	0.19–0.41	0.37 (0.05)	0.25–0.47	0.45 (0.05)	0.36–0.54
Pregnancy glucose concentrations (one-hour post-GTT (mg/dL)	269	124.46 (30.88)	52.00–244.00	131.71 (31.62)	92.00–177.00	118.69 (27.07)	66.00–186.00	126.00 (32.36)	52.00–227.00	149.42 (33.75)	96.0–244.00
Gestational age at GTT (weeks)	269	26.80 (2.56)	8.14–32.57	28.37 (1.94)	24.71–30.29	26.82 (1.59)	23.14–32.14	26.56 (3.68)	8.14–32.57	26.72 (3.55)	16.57–31.86
Pregnancy DII	276	− 1.40 (1.65)	− 4.94–3.88	− 1.08 (2.03)	− 3.13–3.04	− 1.70 (1.49)	− 4.94–2.41	-1.07 (1.69)	− 3.98–3.88	-0.57 (1.98)	− 3.70–3.14
Food desert severity^b^	296	0.13 (0.11)	0.00–0.43	0.15 (0.10)	0.05–0.32	0.12 (0.11)	0.00–0.43	0.15 (0.10)	0.00–0.42	0.17 (0.12)	0.00–0.43
Age (years)	302	31.94 (4.23)	17.84–40.28	33.66 (3.54)	28.20–38.21	32.21 (4.19)	17.84–40.20	31.50 (3.97)	24.13–40.28	31.41 (5.13)	20.54–39.81
Multiparous^c^	282	41.8%	*n* = 118	50.0%		40.8%		39.4%		48.5%	
Racial/ethnic minority status^d^	302	26.8%	*n* = 81	14.3%		24.3%		31.4%		35.3%	
Alcohol use after pregnancy was known^e^	302	9.3%	*n* = 28	0.0%		9.9%		8.6%		8.8%	
Prenatal vitamin use^f^	270	97.0%	*n* = 262	100.0%		97.6%		93.1%		100.0%	
Total household income^g^	272	8.31 (1.93)	1.00–11.00	9.00 (1.63)	6.00–11.00	8.61 (1.79)	1.00–11.00	7.78 (1.97)	2.00–11.00	7.80 (2.12)	2.00–11.00
Household size	282	2.81 (1.13)	1.00–8.00	2.57 (0.79)	2.00–4.00	2.81 (1.19)	1.00–8.00	2.73 (1.05)	2.00–7.00	2.93 (1.02)	2.00–5.00
Years of completed education	284	15.99 (2.56)	9.00–20.00	17.14 (2.73)	13.00–20.00	16.47 (2.50)	9.00–20.00	15.32 (2.48)	9.00–20.00	14.41 (2.14)	9.00–19.00
Reserve savings after debts are paid^h^	243	4.11 (2.85)	1.00–9.00	4.75 (3.30)	1.00–9.00	4.58 (2.81)	1.00–9.00	3.15 (2.57)	1.00–9.00	3.31 (2.95)	1.00–9.00

*GTT* Oral Glucose Tolerance Test (higher scores indicate poorer glucose regulation), *DII* Dietary Inflammatory Index (higher scores indicate consumption of a more pro-inflammatory diet), *LMP* last menstrual period. Higher food desert severity scores indicate that a greater percentage of individuals living in a census tract characterized by lower income and lower access to healthy and affordable foods.

^a^Pregravid weight status is based on pregravid body mass index scores (Underweight: below 18.5, Healthy Weight: 18.5–24.9, Overweight: 25.0–29.9, Obese: 30 and greater).

^b^Percent of census tract living in a food desert.

^c^0 = Primiparous and 1 = Multiparous.

^d^0 = White, non-Hispanic and 1 = Racial/ethnic minority. Participants self-identified as Asian (*n* = 24), Black (*n* = 4), Multiracial (*n* = 32), Native American (*n* = 2), Pacific Islander (*n* = 1), White (*n* = 234), Other (*n* = 3), or Prefer not to answer (*n* = 2). Participants also self-identified as Hispanic (*n* = 25), non-Hispanic (*n* = 276), or Prefer not to answer (*n* = 1).

^e^0 = Did not use alcohol after pregnancy was known and 1 = Used alcohol after pregnancy was known.

^f^0 = Did not use prenatal vitamins and 1 = Used prenatal vitamins.

^g^1 = Less than $5000, 2 = $5000 to $11,999, 3 = $12,000 to $15,999, 4 = $16,000 to $24,999, 5 = $25,000 to $34,999, 6 = $35,000 to $49,999, 7 = $50,000 to $74,999, 8 = $75,000 to $99,999, 9 = $100,000 to $199,999, 10 = $200,000 to $299,999, 11 = $300,000 and greater.

^h^1 = Less than $500, 2 = $500 to $4999, 3 = $5000 to $9999, 4 = $10,000 to $19,999, 5 = $20,000 to $49,999, 6 = $50,000 to $99,999, 7 = $100,000 to $199,999, 8 = $200,000 to $499,999, 9 = $500,000 and greater.

### Bivariate correlations

Bivariate correlations among the focal variables (Table 2) suggest that higher food desert severity scores were associated with greater adiposity (*r* = 0.17; *p* = 0.02) and lower SES (years of education: *r* = − 0.15, *p* = 0.01). Variables related to lower SES were associated with greater adiposity (total household income: *r* = − 0.20, *p* = 0.01; reserve savings after debts are paid: *r* = − 0.17, *p* = 0.04) and with pro-inflammatory dietary intake (total household income: *r* = − 0.15, *p* = 0.02), but not with glucose concentrations. Adiposity, glucose concentrations, and DII scores were all positively correlated (*r*s > 0.21; *p*s < 0.001).

**Table 2:** Bivariate correlations for study variables.

Variable	1	2	3	4	5	6	7	8	9	10	11	12
Total household income^a^	–											
Years of completed education	**0.185**	–										
Reserve savings after debts are paid^b^	**0.323**	**0.295**	–									
Food desert severity^c^	− 0.019	− **0.152**	− 0.121	–								
Pregnancy adiposity (% body fat)	− **0.196**	− 0.132	− **0.165**	**0.174**	–							
Pregnancy glucose concentrations (one-hour post-GTT [mg/dL])	− 0.008	− 0.034	− 0.066	0.096	**0.432**	–						
Gestational age at GTT (weeks)	− 0.066	− 0.069	− 0.009	0.058	− 0.078	− 0.111	–					
Pregnancy DII	− **0.149**	− 0.097	− 0.068	0.034	**0.258**	**0.210**	0.055	–				
Age (years)	**0.138**	**0.343**	**0.251**	− 0.069	− 0.041	0.015	0.040	− **0.159**	–			
Multiparous^d^	− **0.531**	− 0.001	0.010	− 0.021	0.086	− 0.080	0.079	**0.129**	**0.155**	–		
Racial/ethnic minority status^e^	− 0.059	0.006	0.061	0.024	0.129	**0.251**	− 0.088	0.031	− 0.004	− 0.052	–	
Used alcohol after pregnancy was known^f^	0.072	0.030	0.087	**0.122**	− 0.008	0.013	− 0.038	0.097	0.003	− 0.022	− 0.090	–
Used prenatal vitamins^g^	0.107	0.131	0.084	− 0.040	0.073	− 0.081	− 0.012	− 0.069	0.094	0.015	0.102	0.057

*GTT* Oral Glucose Tolerance Test (higher scores indicate poorer glucose regulation), *DII* Dietary Inflammatory Index (higher scores indicate consumption of a more pro-inflammatory diet). Higher food desert severity scores indicate that a greater percentage of individuals living in a census tract have lower income and lower access to healthy and affordable foods.

^a^Total household income adjusted for household size.

^b^1 = Less than $500, 2 = $500 to $4999, 3 = $5000 to $9999, 4 = $10,000 to $19,999, 5 = $20,000 to $49,999, 6 = $50,000 to $99,999, 7 = $100,000 to $199,999, 8 = $200,000 to $499,999, 9 = $500,000 and greater.

^c^Percent of census tract living in a food desert.

^d^0 = Primiparous and 1 = Multiparous.

^e^0 = White, non-Hispanic and 1 = Racial/ethnic minority.

^f^0 = Did not use alcohol after pregnancy was known and 1 = Used alcohol after pregnancy was known.

^g^0 = Did not use prenatal vitamins and 1 = Used prenatal vitamins.

Bolded values indicate significance, *p* < 0.05.

Age of birthing parent was positively correlated with variables associated with SES (total household income: *r* = 0.14; *p* = 0.02; years of education: *r* = 0.34; *p* < 0.001; reserve savings after debts are paid: *r* = 0.25; *p* < 0.001) and negatively correlated with DII scores (*r* = − 0.16; *p* = 0.01). Minoritized racial/ethnicity status was positively correlated with glucose concentrations (*r* = 0.25; *p* < 0.001). Multiparity was significantly correlated with total household income (*r* = − 0.53,* p* < 0.001), pro-inflammatory dietary intake (*r* = 0.13,* p* = 0.04), and age (*r* = 0.16,* p* = 0.01). Endorsement of alcohol use after pregnancy was known was positively correlated with food desert severity (*r* = 0.12, *p* = 0.04), and using prenatal vitamins was positively correlated with years of education (*r* = .13, *p* = .03).

### SES measurement model

A latent variable for SES was created using the measures described above. All factor loadings were significant (*p* < 0.0001) and greater than 0.40 in magnitude, confirming that this is an appropriate way to consider these data. The variance of the latent variable was significant (*p* = 0.02), suggesting inter-individual variability in SES scores. See Table 3 for detailed results of the measurement model.

**Table 3:** Measurement model for SES latent variable.

Latent SES variable			
Indicator	β ± SE	95% CI	*p*
Total household income^a^	0.45 ± 0.09	0.28–0.63	0.0001
Years of completed education	0.41 ± 0.09	0.23–0.59	0.0001
Reserve savings after debts are paid^b^	0.72 ± 0.12	0.47–0.96	0.0001

The measurement model for SES was just-identified, meaning that no fit indices were produced, but that model estimates are reliable.

^a^Total household income adjusted for household size.

^b^1 = Less than $500, 2 = $500 to $4999, 3 = $5000 to $9999, 4 = $10,000 to $19,999, 5 = $20,000 to $49,999, 6 = $50,000 to $99,999, 7 = $100,000 to $199,999, 8 = $200,000 to $499,999, 9 = $500,000 and greater.

### Regressions used to test hypotheses 1 and 2

Table 4 reports the results of the regressions used to test Hypotheses 1 and 2. Consistent with the results of the bivariate correlations, lower SES was associated with greater adiposity (β = − 0.27, *p* = 0.016), greater dietary inflammation (β = − 0.25, *p* = 0.005), and greater food desert severity scores (β = − 0.20, *p* = 0.008). Greater food desert severity scores were associated with greater adiposity (β = 0.17, *p* = 0.013) and glucose concentrations (β = 0.10, *p* = 0.045).

**Table 4:** Effect sizes and significance of the main effects regression models.

Dependent variable	Latent SES variable			Food desert severity		
	β ± SE	95% CI	*p*	β ± SE	95% CI	*p*
Pregnancy adiposity (% body fat)	− 0.27 ± 0.11	− 0.49 to − 0.05	0.016	0.17 ± 0.07	0.04–0.31	0.013
Pregnancy glucose concentrations (one-hour post GTT [mg/dL])	− 0.04 ± 0.10	− 0.24 to 0.12	0.674	0.10 ± 0.05	0.002–0.20	0.045
Pregnancy DII	− 0.25 ± 0.08	− 0.41 to − 0.08	0.003	0.02 ± 0.06	− 0.09–0.14	0.705
Food desert severity	− 0.20 ± 0.07	− 0.34 to − 0.05	0.008	–	–	–

*GTT* oral Glucose Tolerance Test (higher scores indicate poorer glucose regulation), *DII* Dietary Inflammatory Index (higher scores indicate consumption of a more pro-inflammatory diet). Birthing parent age, parity status, racial/ethnic minority status, use of alcohol after pregnancy was known, and use of prenatal vitamins were initially included as covariates in all models. Gestational age at GTT (in weeks) was also included as a covariate in models that included values from the GTT. Covariates that were not significantly associated with pregnancy metabolic variables were dropped from final models to preserve model parsimony. The relationship between SES and food desert severity was only assessed unidirectionally (SES predicting food desert severity).

### SEM used to test hypothesis 3

**Figure 2:**
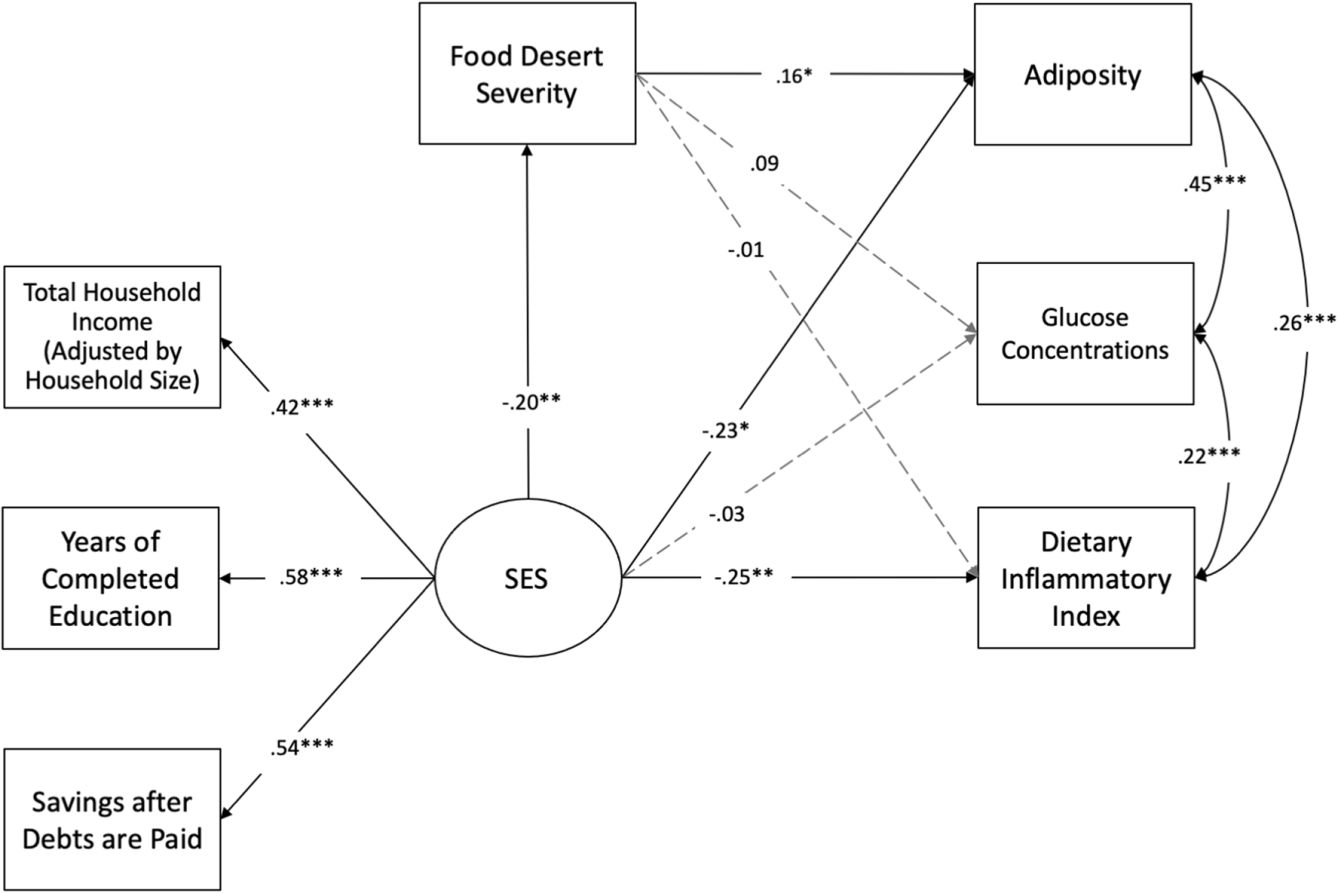
Food Desert Significantly Mediates the Relationship between SES and Adiposity. Food desert severity scores significantly mediated the relationship between latent SES and adiposity (ß_indirect_ = − 0.03, 95% CI [−0.079, −0.004]); after adjusting for age of birthing parent, parity status, and racial/ethnic minority status, as well as for weeks’ gestation at GTT, use of alcohol after pregnancy was known, and use of prenatal vitamins. Food desert severity did not mediate the effect of SES on glucose (95% CI − 0.053, 0.001) or DII scores (95% CI − 0.021, 0.037). *p < 0.05, **p < 0.01, ***p < 0.001. CFI = 0.965; TLI = 0.947; SRMR = 0.039; RMSEA = 0.024, 90% CI [0.000, 0.049], *p* = 0.957^51^.

Figure 2 displays the results of the SEM used to test Hypothesis 3. Lower SES was associated with higher food desert severity scores (β = − 0.20, *p* = 0.007), increased adiposity (β = − 0.23, *p* = 0.030), and higher DII scores (β = − 0.25, *p* = 0.003). SES was not associated with glucose concentrations (*p* = 0.765).

Higher food desert severity scores were associated with greater adiposity (β = 0.16, *p* = 0.036), but not with higher glucose concentrations (*p* = 0.090) or with DII scores (*p* = 0.848). Food desert severity scores significantly mediated the relationship between the latent SES variable and adiposity (β_indirect_ = -0.03, 95% CI [− 0.079, − 0.004]); however, they did not mediate the effect of SES on glucose concentrations (95% CI − 0.053, 0.001) or DII scores (95% CI = − 0.021, 0.037). This model simultaneously tested the relationships between SES and food desert severity and each metabolic variable, adjusting for their interdependence. SES was also associated with age of birthing parent (β = 0.48, *p* = 0.0001), such that higher SES was associated with older ages. Using alcohol after pregnancy was known was positively associated with food desert severity (β = 0.13, *p* = 0.026) and prenatal vitamin use predicted higher adiposity (β = 0.15, *p* = 0.001) and higher SES (β = 0.17, *p* = 0.048). Racial/ethnic minority status significantly predicted glucose concentrations (β = 0.22, *p* = 0.0001), such that individuals from minoritized backgrounds had higher glucose concentrations. Adiposity significantly covaried with glucose concentrations (β = 0.45, *p* = 0.0001) and DII scores (β = 0.25, *p* = 0.0001). Glucose concentrations significantly covaried with DII scores (β = 0.22, *p* = 0.0001). Sensitivity analyses are described in Supplementary Materials.

## Discussion

Metabolic health during pregnancy is an important determinant of health for pregnant people and their infants^1–6,8,10–17^. Low SES is one risk factor for impaired metabolic health during pregnancy^27,28,30^, but the mechanisms through which SES influences metabolic health and nutrition during pregnancy remain relatively underexplored. Here, we assessed whether food desert severity mediated the relationship between SES and adiposity, glucose regulation, and inflammatory level of diet during pregnancy. The findings of this study provide overall support for a relationship of both SES and food desert severity on metabolic health during pregnancy. Results suggest that food desert severity is a mechanism by which SES influences adiposity during pregnancy, such that individuals with lower SES tend to experience more food desert severity, which leads to increasing adiposity during pregnancy.

Consistent with earlier work^28,32–34^, results from main effects models (Table 4) indicated that lower SES was significantly associated with greater adiposity, greater food desert severity, and consumption of a more pro-inflammatory diet. As adiposity, glucose concentrations, and the pro-inflammatory nature of participants’ prenatal diets were all positively and significantly correlated, we account for their interdependence through our model design. It is interesting to note that the pro-inflammatory nature of participants’ prenatal diets was significantly correlated with glucose concentrations. While dietary quality likely impacts glucose regulation and adiposity during pregnancy, it is also possible that overall metabolic state, informed by GTT results and body composition, may impact diet. For example, an individual at-risk for GDM may alter their diets in response to their GTT results or at the instruction of their physician, particularly if their physician recommends trying to limit pregnancy weight gain (as is often the case for individuals with overweight or obesity)^68^. After adjusting for adiposity, glucose concentrations, and dietary inflammation, as well as the interdependence of these variables and for relevant covariates, the effect of SES on food desert severity survived.

We also found that higher food desert severity was associated with higher adiposity and poorer glucose regulation during pregnancy (Table 4). The effect of higher food desert severity associated with increasing adiposity during pregnancy survived accounting for the effect of SES, the interdependence of metabolic health measures, and relevant covariates (Fig. 2). These findings are consistent with work in non-obstetric samples showing that living in a food desert is associated with obesity^19^. While living in a food desert is certainly related to SES, our results indicate that they are not equivalent constructs or metrics. For example, it is possible that some individuals living in a food desert may have the financial resources necessary to access healthful foods or vice versa. Results from our study suggest that, after accounting for food desert severity, SES is not independently related to glucose concentrations during pregnancy (Fig. 2). Our findings also suggest that living in a food desert is not associated with increased circulating glucose concentrations in response to the GTT, which is consistent with other work suggesting that living in a food desert is not associated with increased GDM risk^69^, but in contrast to work suggesting reduced GDM risk in areas with more grocery stores^22^. We posit that these discrepancies may be due, in part, because past work has not simultaneously accounted for the interdependent effects of SES and food desert severity.

Food desert severity scores significantly mediated the relationship between lower SES and greater adiposity, even after accounting for the pro-inflammatory quality of the participants’ diet, their glucose concentrations during pregnancy, and relevant covariates (Fig. 2). This important finding offers a mechanistic explanation of *how* SES impacts metabolic health during pregnancy. Consistent with our initial hypotheses, lower SES was associated with greater food desert severity scores (likely because SES may influence the types of neighborhoods in which one can afford to live), and food desert severity, in turn, was associated with increased adiposity during pregnancy. This is the first study to our knowledge to simultaneously assess the effects of food desert severity and SES on adiposity, glucose concentrations, and dietary inflammation during pregnancy. Notably, the effect of SES on adiposity via food desert severity was independent of the inflammatory nature of diet, which was not associated with food desert severity in this model. This is in contrast to other work in non-obstetric samples showing that living in a food desert is associated with an unhealthy diet (i.e., typically measured by lower fruit and vegetable consumption)^46^. Future research should consider other mechanisms through which food desert severity may impact pregnancy adiposity. For example, individuals living in food deserts may experience chronic stress associated with poverty, which may be independently associated with dysregulated gestational weight gain^39^.

It is possible that measuring specific micro- or macro- nutrients in the dietary intake of pregnant individuals living food deserts, rather than the overall pro-inflammatory quality of their diets, may be more informative; however, we did not find that either total caloric intake or percent of total calories from fat during the second trimester had a significant relationship with SES or food desert severity (see Supplementary Figs. S5–S6). It is also possible that physical activity contributes to this relationship; however, we did not find that self-reported physical activity during pregnancy (as measured by the Active Living Index, calculated from the Kaiser Physical Activity questionnaire^70^) explained our findings (See Supplementary Fig. S7). Thus, future work may benefit from targeting other differences in specific nutrient consumption by pregnant populations, as well as from exploring the influence of physical activity on pregnancy adiposity further. We note that our physical activity measure was self-reported physical activity during pregnancy, rather than a more robust objective measurement of physical activity during pregnancy, such as via accelerometry.

This study had a number of strengths. To our knowledge, this is the first study to examine whether food desert severity mediates the relationship between low SES and poor metabolic health during pregnancy. Whereas most other studies of food deserts utilize a dichotomous assessment of food desert status^43^, in this study, we utilize a continuous measure of food desert severity. The continuous measure of food desert severity allowed for quantification of the linear association between food desert severity and metabolic health and may be useful for drawing inferences relevant to individuals in low-income, low access areas, but who do not meet the threshold for living in a food desert, as well as for individuals who do not reside in food deserts, but have lower access to healthy food. This study was also strengthened by the detailed characterization of participants’ diets via repeated 24-h recalls conducted by registered nutritionists (vs. participant report, as has been utilized in most previous research), which may have increased the reliability of the dietary information. Moreover, rather than using self-reported weight gain, we utilized air displacement plethysmography to assess body composition, allowing us to distinguish between the percent of fat and lean mass during pregnancy.

### Limitations and suggestions for future research

This study also suggests several directions for future study. Results suggest that factors beyond the pro-inflammatory quality of the diet may be responsible for the relationship between higher food desert severity and greater adiposity during pregnancy. Future studies should assess other factors relevant to the metabolic health of those living in food deserts. For example, neighborhoods with low access to healthful and affordable foods may also have poor incorporation of features that encourage physical activity such as sidewalks and greenspaces^71^. Neighborhoods with higher crime rates may also be associated with lower physical activity due to safety concerns^72^. Physical activity during pregnancy alone is unlikely to explain these findings, however, as analyses conducted with self-reported physical activity as an outcome measure did not show a relationship between physical activity during pregnancy and SES or food desert severity (See Supplementary Fig. S7). Furthermore, food deserts may have more pollution, which can contribute to poor cardiometabolic health^73^. Due to our specific interest in mechanisms associated with metabolic health during pregnancy, we focused on adiposity during pregnancy in our primary models (rather than utilizing a measure of pre-gravid BMI, which may also be related to SES and food desert severity as well as health complications before, during, and after pregnancy^74^). This focus allowed us to temporally align our various measures of metabolic health and to control for their interdependence, an approach that strengthened our ability to make inferences relevant to this unique developmental period. However, it is important to note that the effects of SES and food desert severity are not likely to be specific to pregnancy and may predate pregnancy and/or persist after birth. It remains clear that, while variation in SES and food desert severity may not be specific to understanding metabolic health only during pregnancy, SES and food desert severity contribute unique importance to identifying risk for poor metabolic health during pregnancy, a developmental period with long-term significance for the parent and child. Furthermore, while geospatial coding is a novel means of collecting objective data about the food desert severity, there are some limitations associated with this technique. For example, individuals within the same census tract may have different access to healthful foods, depending on where in the tract they reside. Although food desert severity provides a sense of proximity to healthful foods (and thus potential difficulty in obtaining these foods), this is, of course, not deterministic. Future research should explore other factors related to food access. For example, individuals may live in a food desert, but have access to healthful foods via grocery delivery or other means. Future studies may benefit from triangulating access to healthy and affordable foods via multi-modal data collection, including geospatial coding and surveys concerning accessibility to healthful and affordable foods. Given the growing literature on “food swamps”, areas where convenience stores and fast-food restaurants outnumber retailers with healthier options (e.g., grocery stores), the relationship between food swamp severity and pregnancy metabolic health should also be assessed. An additional and important limitation of this study is that the USDA Food Access Research Atlas data from which the food desert measures were calculated utilized data from the 2010 Census of the Population. The pregnancies in our study occurred between 2018 and 2021. It is possible that census tracts may have changed since 2010. Additionally, average incomes in particular census tracts may have changed between 2014 and 2019 and new supermarkets may have been constructed between 2018 and 2019. Thus, areas that were considered food deserts based off of these earlier data sources may no longer be considered food deserts. While these were the most up-to-date UDSA FARA data available at the time of this study, future studies should utilize updated USDA FARA data when it is released. Finally, while our sample characteristics are consistent with the demographics of the Portland, Oregon metropolis, the majority of our participants were white, non-Hispanic, and of higher SES. Future research should validate these findings in a larger more diverse sample that is more representative of the United States population.

## Conclusions

The finding that food desert severity significantly mediates the association between low SES and adiposity during pregnancy is an important step toward identifying targets for interventions aimed at improving metabolic health during pregnancy, and, by extension, offspring birth outcomes and long-term health. Identifying food desert severity as a mechanism by which SES impacts adiposity during pregnancy lends important credence for supporting certain interventions aimed at improving metabolic health during pregnancy over others. For example, improving access to healthful and nutritious foods may be a step toward improving metabolic health during pregnancy for groups at increased risk for adiposity (i.e., low SES individuals), which may, in turn, improve offspring outcomes. As individuals with higher percent adiposity during pregnancy may not have exceeded clinical cutoffs for unhealthy pre-gravid BMIs^68^, understanding the mechanisms responsible for increased body fat during pregnancy may be a useful means of identifying which individuals may be most liable for metabolic risk during pregnancy. Results indicate a critical need to focus on making healthful and affordable foods accessible in all communities. This can be implemented via micro-, mezzo-, and macro-interventions, which may include providing community gardens or encouraging healthful dietary habits via training/education on food preparation and nutrition (micro), enrolling eligible residents into federal supplemental nutrition assistance programs or encouraging participation in food system planning (mezzo), or enacting legislation and policies to incentivize the addition of full-service grocery stores and supermarkets to underserved communities and improving the quality of food available at existing convenience stores (macro).

## Supplementary Information

Supplementary Information.

## Data Availability

The data set described in this manuscript will be publicly available on the National Institute of Mental Health (NIMH) Data Archive (https://nda.nih.gov/) under study #2069, DOI: 10.15154/1528786. The study page provides instructions on locating the data.
