# Supplementary material for: The association between food desert severity, socioeconomic status, and metabolic state during pregnancy in a prospective longitudinal cohort
Source: Sci Rep. 2023 May 3;13:7197. doi: 10.1038/s41598-023-32783-2 (PMC10156695; doi:10.1038/s41598-023-32783-2)
Supplement: Supplementary file 1 — Supplementary Information. [file 41598_2023_32783_MOESM1_ESM.docx]

SUPPLEMENTARY MATERIALS

**The association between food desert severity, socioeconomic status, and metabolic state during pregnancy in a prospective longitudinal cohort**

Elizabeth K. Wood, PhD^a^, Gayle Stamos, BS^a^, AJ Mitchell, PhD^b^, Rose Gonoud, BS^a^, Angela M. Horgan, PhD^c^, Olivia Nomura, BA^a^, Anna Young, BSN^a^, Joel T. Nigg, PhD^a^, Hanna C. Gustafsson, PhD^a^, Elinor L. Sullivan, PhD^a,b,d^

^a^ Department of Psychiatry, Oregon Health & Science University

^b^ Department of Behavioral Neuroscience, Oregon Health & Science University

^c^ Clinical & Translational Research Center, Oregon Health & Science University

^d^ Division of Neuroscience, Oregon National Primate Research Center

Keywords: adiposity, dietary inflammatory index, glucose regulation, maternal metabolic health, socioeconomic status

Corresponding Author:

Elinor L. Sullivan

Department of Psychiatry

Oregon Health & Science University

Mailcode: L470

3181 SW Sam Jackson Park Road

Portland, OR 97239-3098

503-346-5516

Supplementary Methods

*Recruitment & Exclusionary Criteria*

Patients receiving prenatal care through OHSU and its affiliated clinics were identified via electronic medical records and were contacted via email or telephone or in-person at prenatal intake appointments to assess interest and eligibility. When available, medical records were reviewed for exclusionary conditions prior to contact, to minimize participant and staff burden. Participants were also recruited via fliers located on the OHSU campus and via social media advertisements targeting people living in the greater Portland metropolitan area. All participants were screened for eligibility via telephone. Exclusionary criteria included being pregnant with multiples, known fetal anomaly or genetic condition that may influence child brain development or behavior, current substance use (illicit drugs, tobacco, marijuana), and current use of medications that may influence inflammation (e.g., systemic corticosteroids) or that have known or suspected teratogenic effects. Individuals who had consumed 10 or more alcoholic drinks during pregnancy at the time of enrollment were not eligible to participate in the study. Individuals with a history of recurrent pregnancy loss in the second or third trimester were also excluded. Medical conditions that might affect inflammation or be confounded with obesity were also exclusionary criteria, including a current diagnosis of Diabetes Type I/II, Cancer, Kidney Disease, Polycystic Ovarian Syndrome (as confirmed by hyperandrogenism or a history of medication used to treat the condition), and autoimmune diseases. Furthermore, individuals with a history of bariatric surgeries were excluded from the study. In recruiting, prioritization was given to patients who planned to give birth at OHSU, to facilitate the collection of delivery tissue.

In the overall study, 304 participants were followed across 309 pregnancies (five participants were followed across two pregnancies). Participants were excluded from the present study for reasons that may have impacted the findings, including: participant diagnoses with an autoimmune disease during pregnancy (*n* = 1), miscarriages or stillbirths (*n* = 3), infant born with previously undiagnosed genetic disorders or major health complications (*n* = 2), and infant born extremely preterm (i.e., less than 28 weeks; *n* = 1), yielding a final analytic sample of 302 pregnancies (see Supplementary Fig. S1 for a flow chart).

Supplementary Figure S1.
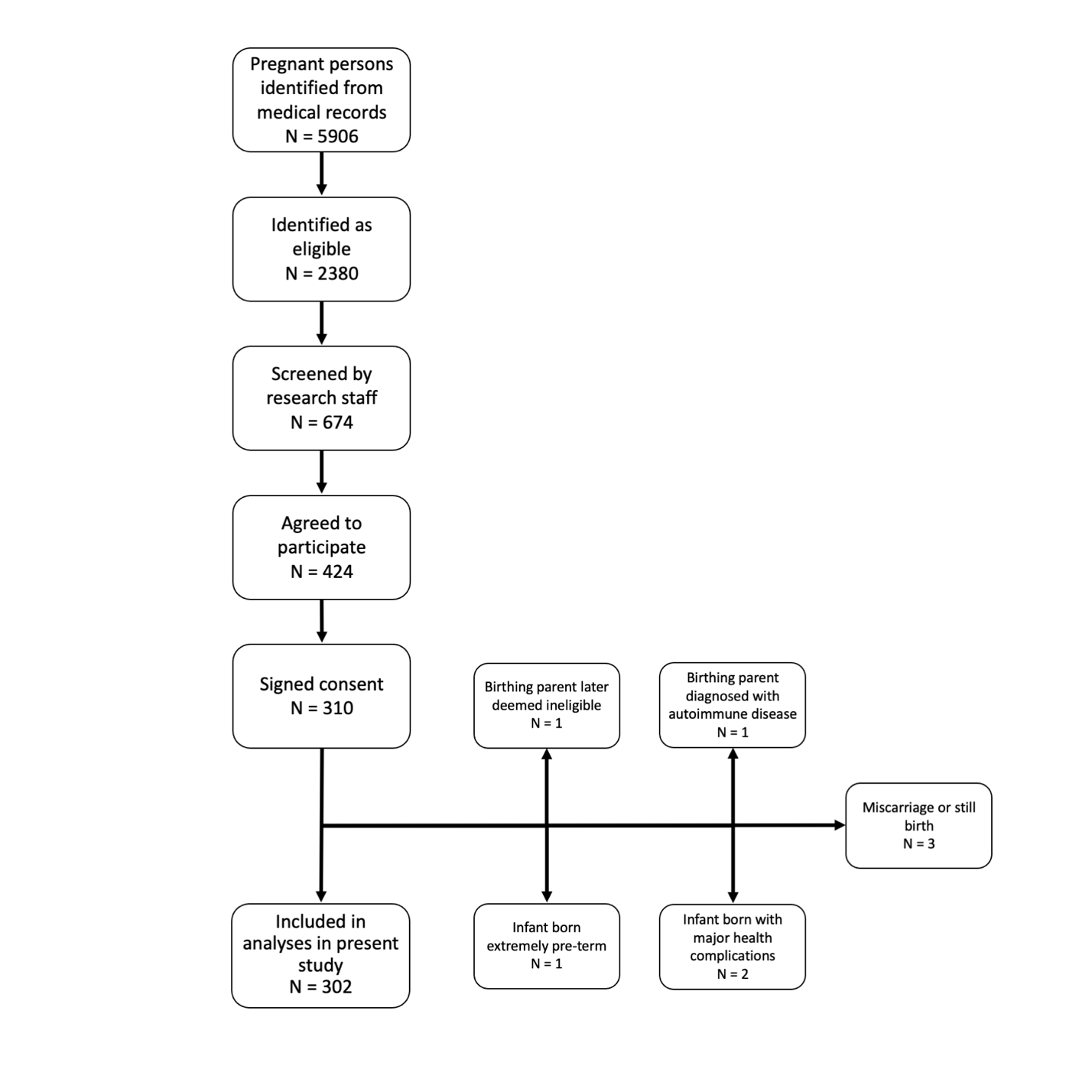


*Supplementary Figure S1 Caption.* Flow chart depicting recruitment and eligibility for the overall study, as well as reasons that participants that were excluded from the analyses for the present study.

*Glucose Regulation*

While the majority of participants for whom there were glucose tolerance test (GTT) data completed the GTT between 24 and 28 weeks’ gestation (*n* = 230) (the time frame recommended by the American College of Obstetrics and Gynecology for routine surveillance), there were some participants who completed the test earlier (*n* = 8) or later in gestation (*n* = 31) due to risk factors related to the acquisition of gestational diabetes, scheduling issues, travel, or due to challenges related to the COVID-19 pandemic. To account for this variability, the participant’s gestational age at the time of the GTT was included as a covariate in all analyses that assessed GTT data. As some participants completed multiple GTTs (*n* = 22), we first opted to include the values for tests that occurred between the 24-to-28 weeks’ gestation window. Second, we prioritized the inclusion of test results that included more complete data (e.g., those that included fasting glucose values, 1-hour glucose values, and 2-hour glucose values, vs. a single assessment of glucose).

*Adiposity*

Adiposity was assessed using air displacement plethysmography via the BOD POD^®^ Body Composition tracking system (Life Measurement, Inc.). Briefly, participants changed into skin-tight clothing (i.e., swimming suit or sports bra and spandex shorts) and wore a swimming cap. They sat inside the BOD POD^®^ chamber and whole-body densitometry was used to determine the amount of fat and lean tissue in the body, via the displacement of air. Following standard procedures, two 60-second measures were collected during both the second trimester of pregnancy and were averaged. Participants were instructed to fast overnight and in the morning prior to testing and to not exercise for three-hours prior to testing. The procedure took approximately 15 minutes.

*Nutrition*

Trained staff, overseen by a dietitian certified in the procedure, used the multi-pass method to record participant food and drink intake during the previous day. The NDSR software was developed by the Nutrition Coordinating Center (versions 2018-2020; University of Minnesota, Minneapolis, Minnesota) and facilitates the collection of recalls in a standardized fashion using a multiple-pass interview approach. The recalls were unscheduled and unannounced and took place over a two-week period and included the food and drink consumed during one weekend day and two weekdays. Macro- and micronutrient intake across the three days were averaged to create an estimate of dietary intake. All interviewers completed a training program and met qualification standards established in the Oregon Clinical and Translational Research Institute Bionutrition Unit for the NDSR software.

*Dietary Inflammatory Index*

Briefly, inflammatory effect scores of each food parameter (including total energy, macronutrients, micronutrients and specific foods and seasonings) were derived and then standardized to a world database, from which global means and standard deviations were derived. The global mean value for each food parameter was subtracted from each participant’s actual intake value for each food parameter and then divided by the global standard deviation to create a z-score. The z-scores were then multiplied by the inflammatory effect score for each parameter. An overall DII score was derived by summing the inflammatory effect scores for each food parameter.

*Socioeconomic Status*

Participants self-reported information about their SES, including years of education completed (“What is the highest year of regular school that you have completed?”, options ranged from 1-20 years, with high school beginning at 9 years, college beginning at 13 years, and professional/graduate school beginning at 17 years), total annual combined household income (“Which of these categories best describes your total combined family income for the past 12 months?), and amount of savings after adjusting for debt (“Suppose you needed money quickly, and you cashed in all of your (and your spouse’s, if applicable) checking and savings accounts and any stocks and bonds. If you subtracted out any debt that you have (credit card debt, unpaid loans, including car loans), about how much would you have left?”. Total combined family income was categorized as follows: 1 = Less than $5,000, 2 = $5,000 through $11,999, 3 = $12,000 - $15,999, 4 = $16 000 through $24,999, 5 = $25,000 through $34,999, 6 = $35,000 through $49,999, 7 = $50,000 through $74,999, 8 = $75,000 through $99,999, 9 = $100,000 through $199,999, 10 = $200,000 through $299,999, 11 = $300,000 and greater. Total combined family income was adjusted for the number of people residing in the household (“How many people are currently living in your household, including yourself?”). Total savings after debt was categorized as follows: 1 = Less than $500, 2 = $500 to $4,999, 3 = $5,000 to $9,999, 4 = $10,000 to $19,999, 5 = $20,000 to $49,999, 6 = $50,000 to $99,999, 7 = $100,000 to $199,999, 8 = $200,000 to $499,999, 9 = $500,000 and greater.

*Food Desert Severity*

The most-recent version of the United States Department of Agriculture (USDA) Food Access Research Atlas (FARA) utilizes population data from the 2010 Census of the Population. Briefly, participants’ addresses at study enrollment were converted to latitudes and longitude coordinates and were downloaded at the census-block level and then allocated to 0.5-kilometer-square grid cells. To assess distance to the nearest grocery store, the entire country was geographically divided into 0.5-km square grids and then the distance to the nearest grocery store was measured for each grid cell. A list of supermarkets, supercenters, and large grocery stores generated from two independent directories of stores –TDLinx and from stores authorized to accept Supplemental Nutritional Assistance Program (SNAP) benefits was used to calculate the distance to the nearest grocery store for each grid cell by calculating the distance between the geographic center of the 0.5-km square grid that contains estimates of the population and the center of the grid with the nearest grocery store, supercenter, or supermarket. Grocery stores, supercenters, and supermarkets are considered healthful food providers because they sell fresh foods, including fresh fruits and vegetables. These differ from convenience stores, which tend to offer a limited variety of foods that constitute a healthy diet.

*Covariates*

*Participant Age*

Participant age at their last menstrual period was determined by comparing the participant’s date of birth with their estimated delivery date (EDD). Date of birth and EDD were confirmed via electronic medical record review.

*Parity*

Parity was recorded from participants’ electronic health records. Parity values in our sample included 0 (*n* = 163), 1 (*n* = 87), 2 (*n* = 23), 3 (*n* = 6), and 4 (*n* = 2), or Unknown (*n* = 21). Given the low rates of participants who had given birth more than once prior to study enrollment, parity was categorized into primiparous (*n* = 163) and multiparous (*n* = 118), or unknown (*n* = 21) for use in analysis.

*Race/Ethnicity*

Race and ethnicity were reported by participants in the third trimester or were determined via medical record review. Participants identified as Asian (*n* = 24), Black (*n* = 4), Multiracial (*n* = 32), Native American (*n* = 2), Pacific Islander (*n* = 1), White (*n* = 234), Other (*n* = 3), or Unknown (*n* = 2). Participants also identified as Hispanic (*n* = 25), non-Hispanic (*n* = 276), or Unknown (*n* = 1). Given the low rates of racial/ethnic diversity in our sample, race and ethnicity were combined and categorized into White, non-Hispanic *(n =* 219), Racial/Ethnic Minority *(n =* 81), or Unknown (*n* = 2).

*Alcohol Use During Pregnancy*

Participants self-reported whether or not they used alcohol after their pregnancy was known at study enrollment and in the third trimester (Yes = 28; No = 274).

*Use of Prenatal Vitamins*

Participants self-reported whether or not they supplemented with prenatal vitamins in the second and third trimesters of pregnancy (Yes = 262, No = 8, Did not answer = 32 participants).

Supplemental Analyses—Sensitivity Analyses

*Model Examining Varying Timing of Glucose Tolerance Testing*

As described above, there was some heterogeneity as to when in pregnancy the GTT test was completed (range = 8.14-32.57 weeks). To examine whether our study’s findings were biased by values obtained outside of the ACOG recommended time frame (24-28 weeks gestation), we re-ran our primary model without data from these individuals included. The overall direction of the effects remained unchanged when we only included GTT testing results that occurred during the traditional 24-28 window (*n* = 235 participants; see Supplementary Fig. 2); however, as some of the effects became marginal, we covaried weeks’ gestation at GTT testing in all models.

Supplementary Figure S2.


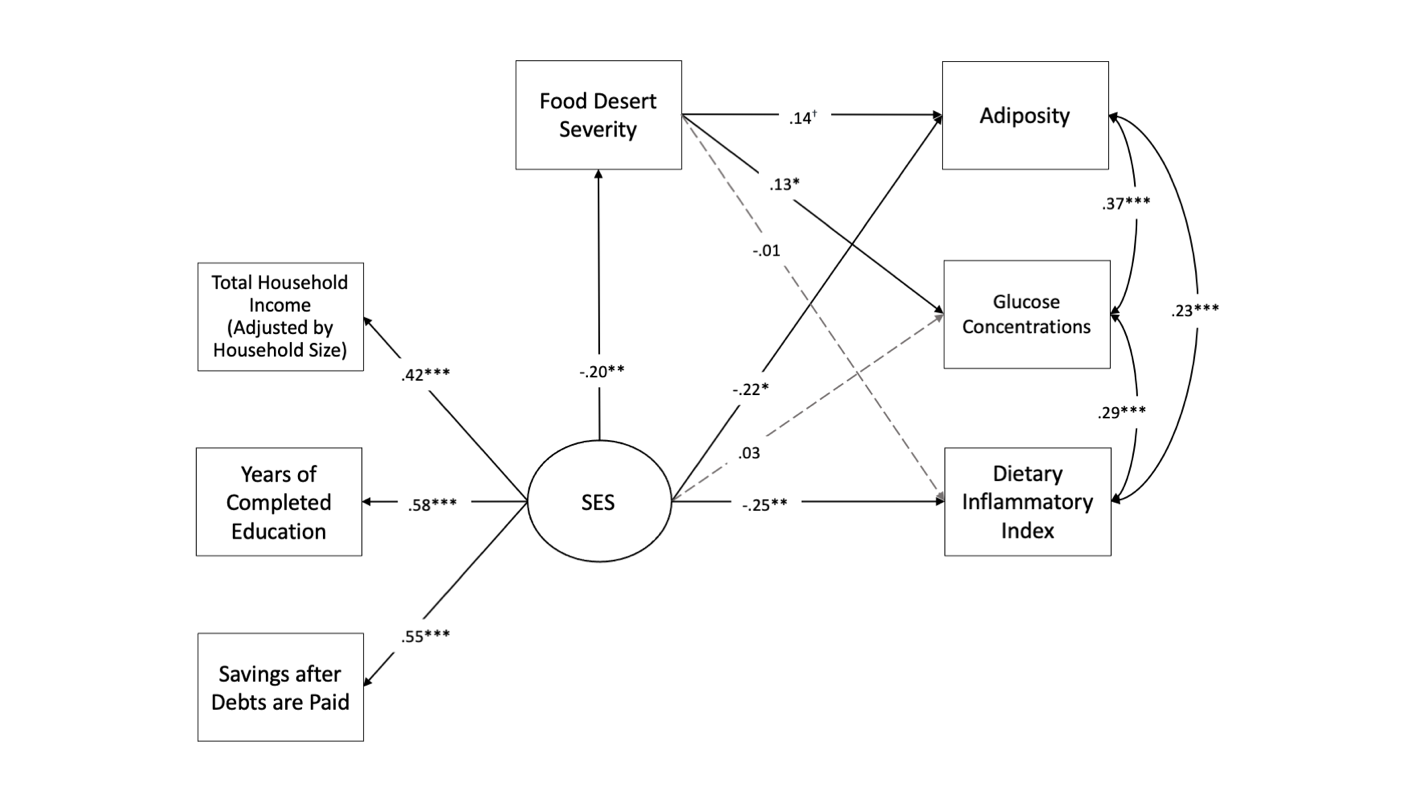


*Supplementary Figure S2 Caption.* Food desert severity scores significantly mediated the relationship between latent SES and adiposity (ß_indirect_= -.03, 95% CI [-.076, -.002]), after adjusting for age of birthing parent, parity status, and racial/ethnic minority status, as well as for weeks’ gestation at GTT, use of alcohol after pregnancy was known, and use of prenatal vitamins. Lower SES was associated with higher food desert severity scores (β = -.20, *p* = .008), higher adiposity (β = -.22, *p* = .048), and consumption of a more pro-inflammatory diet (β = -.25, *p* = .003). Higher food desert severity was associated with higher glucose concentrations (β = .12, *p* = .034) and the association between higher food desert severity scores and higher adiposity was trending (β = .14, *p* = .068). Model statistics: CFI = 0.968; TLI = 0.951; SRMR = .040; RMSEA = .023, 90% CI [.000, .050], *p* = .950*.* ****p* < .001, ***p* < .01, **p* < .05, †*p* < .10

*Model Excluding Participants who met Criteria for Gestational Diabetes Mellitus*

The overall direction of the effects remained unchanged when participants that met criteria for gestational diabetes mellitus (GTT 1h ≥ 180 mg/dL; *n* = 11 participants; 3.64% decrease in sample size) were excluded (see Supplementary Fig. 3).

Supplementary Figure S3.

*
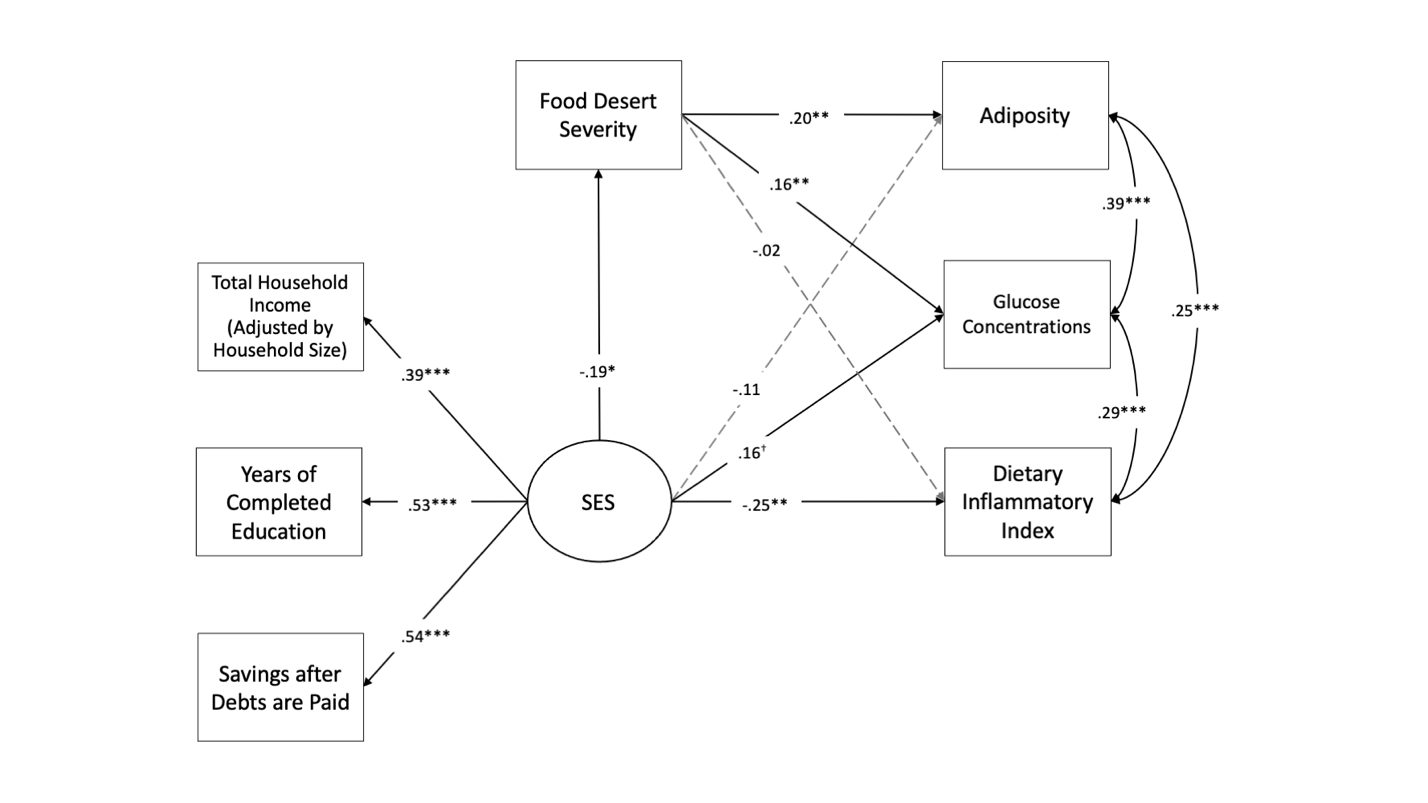
*

*Supplementary Figure S3 Caption.* Food desert severity scores significantly mediated the relationship between latent SES and adiposity (ß_indirect_= -.04, 95% CI [-.078, -.001]), after adjusting for age of birthing parent, parity status, and racial/ethnic minority status, as well as for weeks’ gestation at GTT, use of alcohol after pregnancy was known, and use of prenatal vitamins. Lower SES was associated with higher food desert severity scores (β = -.19, *p* = .014), consumption of a more pro-inflammatory diet (β = -.25, *p* = .007), and the association between higher SES and higher glucose concentrations was trending (β = .16, *p* = .056). There was a significant association between higher food desert severity scores and higher adiposity (β = .20, *p* = .007), as well as between food desert severity and glucose concentrations (β = .16, *p* = .005). Model fit statistics: CFI = 0.954; TLI = 0.930; SRMR = .040; RMSEA = .027, 90% CI [.000 .054], *p* =.915*.* ****p* < .001, ***p* < .01, **p* < .05, †*p* < .10

*Model Excluding Participants who met Criteria for Pre-eclampsia*

The overall direction of the effects remained unchanged when participants that were diagnosed with pre-eclampsia (identified through medical records; *n* = 22 participants, 7.28% decrease in sample size) were excluded (see Supplementary Fig. S4).

Supplementary Figure S4.
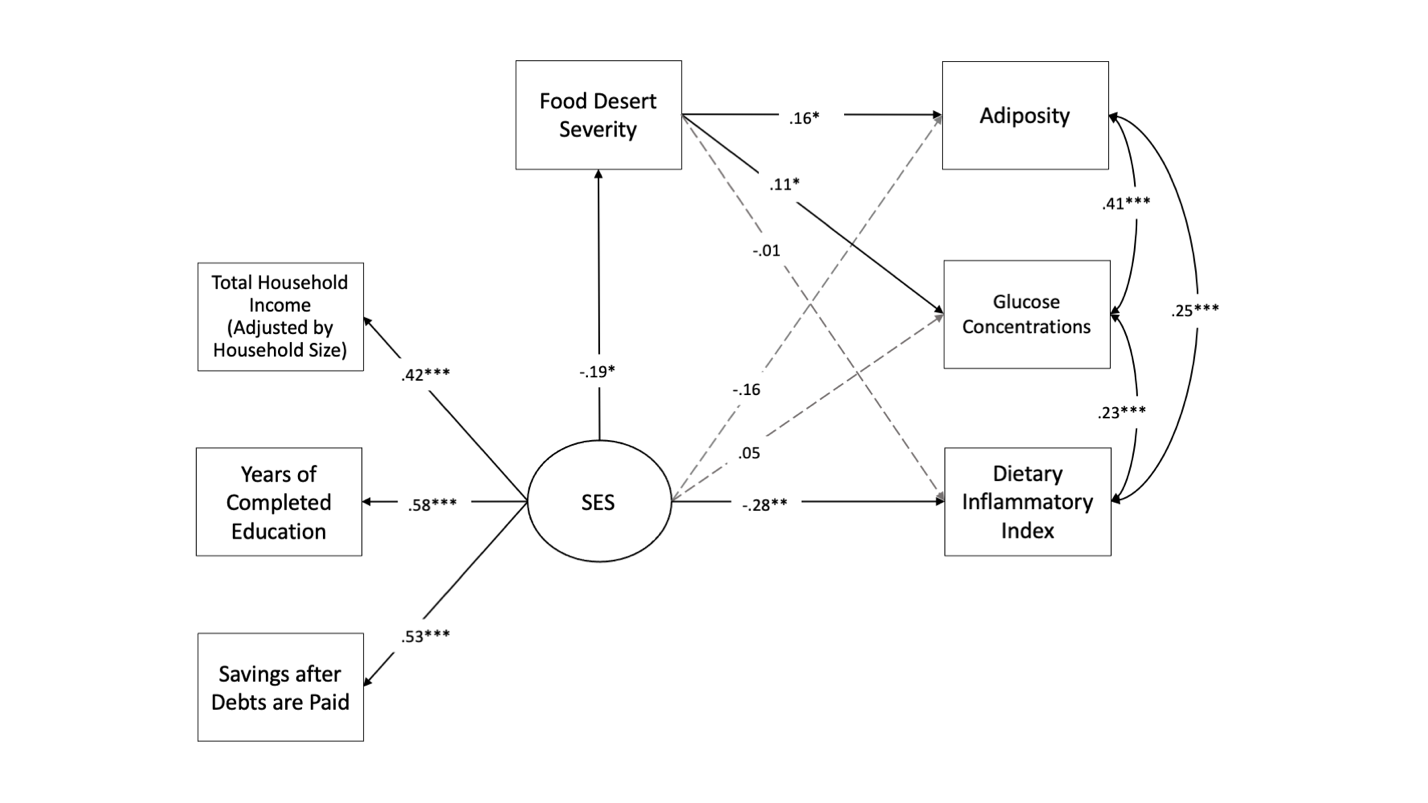


*Supplementary Figure S4 Caption.* Lower SES was associated with higher food desert severity scores (β = -.19, *p* = .019) and consumption of a more pro-inflammatory diet (β = -.28, *p* = .002). There was a significant association between higher food desert severity scores and higher adiposity (β = .16, *p* = .046), as well as between food desert severity and glucose concentrations (β = .11, *p* = .043). Model fit statistics: CFI = 0.946; TLI = 0.918; SRMR = .044; RMSEA = .031, 90% CI [.000 .057], *p* =.878*.* ****p* < .001, ***p* < .01, **p* < .05

*Models Examining Total Calories Consumed and Total Calories from Fat Consumed*

To determine whether food desert severity and SES had other effects of on diet, we examined two models in which average total calories consumed and average percent of total calories from fat consumed in the second trimester were included, respectively. These variables were collected using the same repeated dietary recall methods as described in the main text, conducted by a trained nutritionist. The overall direction of the effect of SES on food desert severity and food desert severity on adiposity remained unchanged; however, there were no significant relationships between SES and dietary measures when total calories or percent of total calories from fat were included in the models (see Supplementary Figs. S5 and S6).

Supplementary Figure S5.


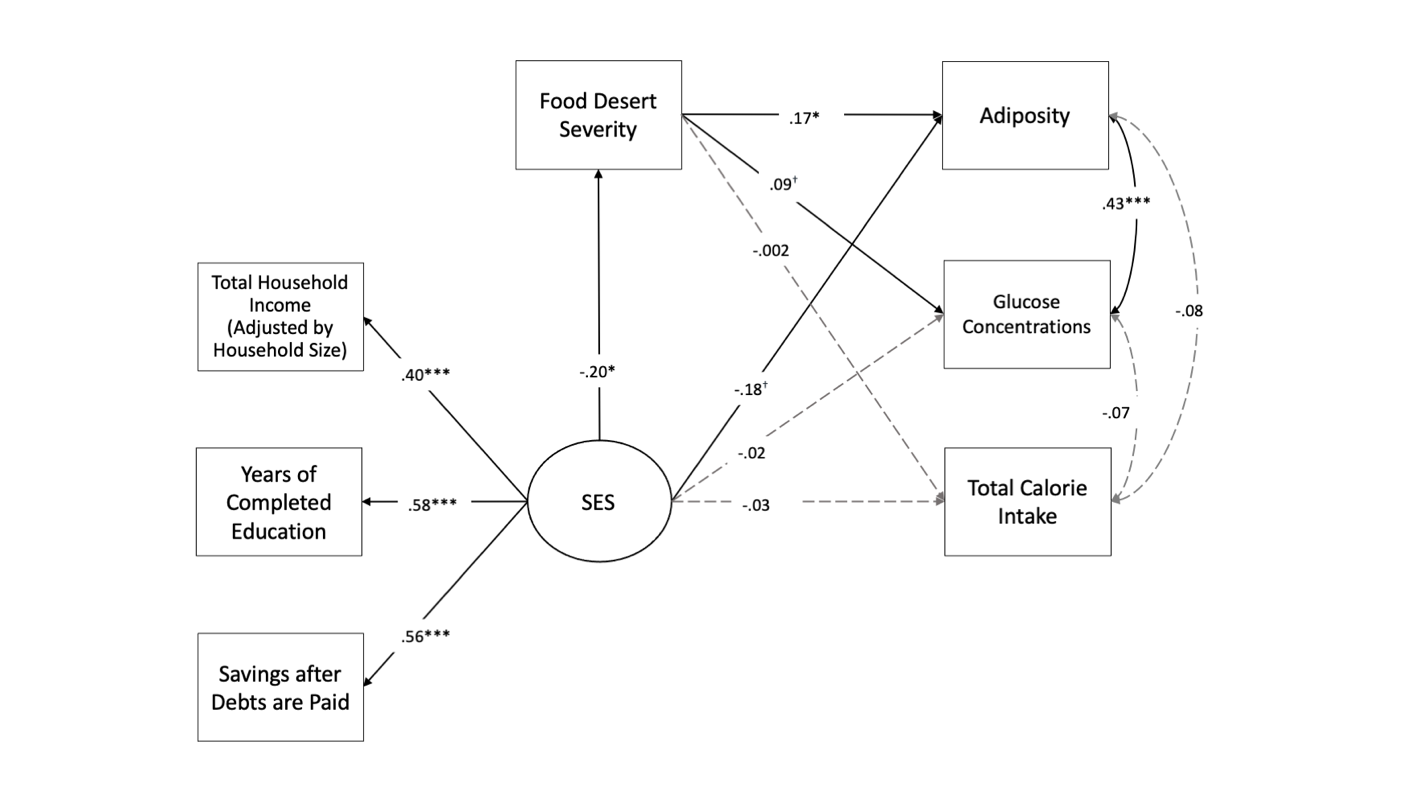


*Supplementary Figure S5 Caption.* Food desert severity scores significantly mediated the relationship between latent SES and adiposity (ß_indirect_= -.03, 95% CI [-.083, -.006]), after adjusting for age of birthing parent, parity status, and racial/ethnic minority status, as well as for weeks’ gestation at GTT, use of alcohol after pregnancy was known, and use of prenatal vitamins. Lower SES was associated with higher food desert severity scores (β = -.20, *p* = .007) and there was a trending relationship between lower SES and higher adiposity (β = -.18, *p* = .097). There was a significant relationship between higher food desert severity scores and higher adiposity (β = .17, *p* = .026) and there was a trending relationship between higher food desert severity and higher glucose concentrations (β = .09, *p* = .095). There was not a significant relationship between total calories consumed and SES (*p* = .753) or food desert severity (*p* = .978). Model fit statistics: CFI = 0.968; TLI = 0.951; SRMR = .039; RMSEA = .022, 90% CI [.000 .050], *p*=.952*.* ****p* < .001, ***p* < .01, **p* < .05, †*p* < .10

Supplementary Figure S6.
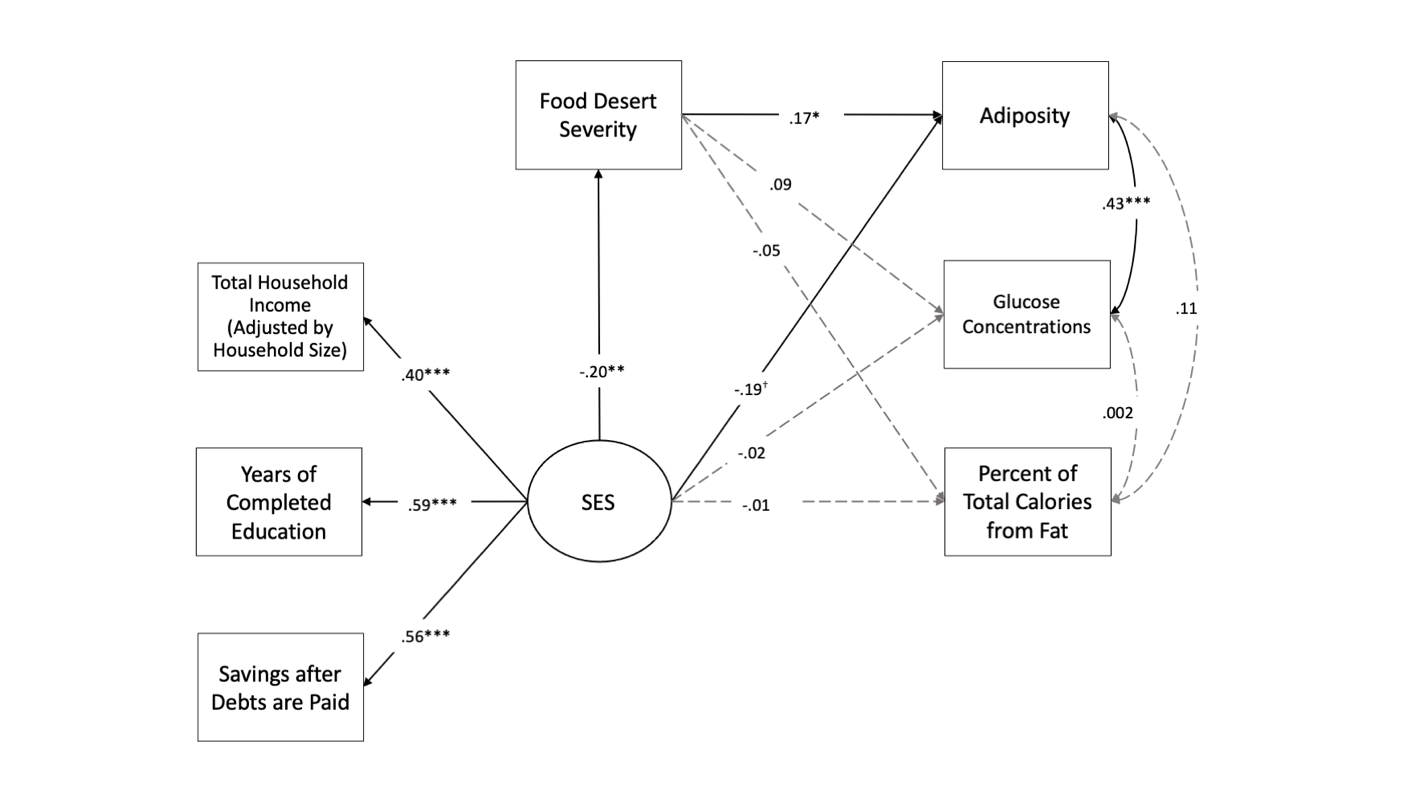


*Supplementary Figure S6 Caption.* Food desert severity scores significantly mediated the relationship between latent SES and adiposity (ß_indirect_= -.03, 95% CI [-.085, -.006]), after adjusting for age of birthing parent, parity status, and racial/ethnic minority status, as well as for weeks’ gestation at GTT, use of alcohol after pregnancy was known, and use of prenatal vitamins. Lower SES was associated with higher food desert severity scores (β = -.20, *p* = .007) and the relationship between higher SES and lower adiposity was trending (β = -.19, *p* = .085). There was a significant relationship between higher food desert severity scores and higher adiposity (β = .17, *p* = .022). There was not a significant relationship between percent of total calories from fat and SES (*p* = .924) or food desert severity (*p* = .468). Model fit statistics: CFI = 0.947; TLI = 0.919; SRMR = .042; RMSEA = .028, 90% CI [.000 .054], *p* = .913*.* ****p* < .001, ***p* < .01, **p* < .05

*Model Examining Activity during Pregnancy*

In an effort to understand the role of food desert severity and SES on activity during pregnancy, we also fit a model that included self-reported physical activity during pregnancy (as measured by the Active Living Index, calculated from the Kaiser Physical Activity questionnaire) as an additional outcome measure. While the previously-seen effects were present, there was no relationship between either SES or food desert severity on self-reported physical activity during pregnancy (see Supplementary Fig. S7).


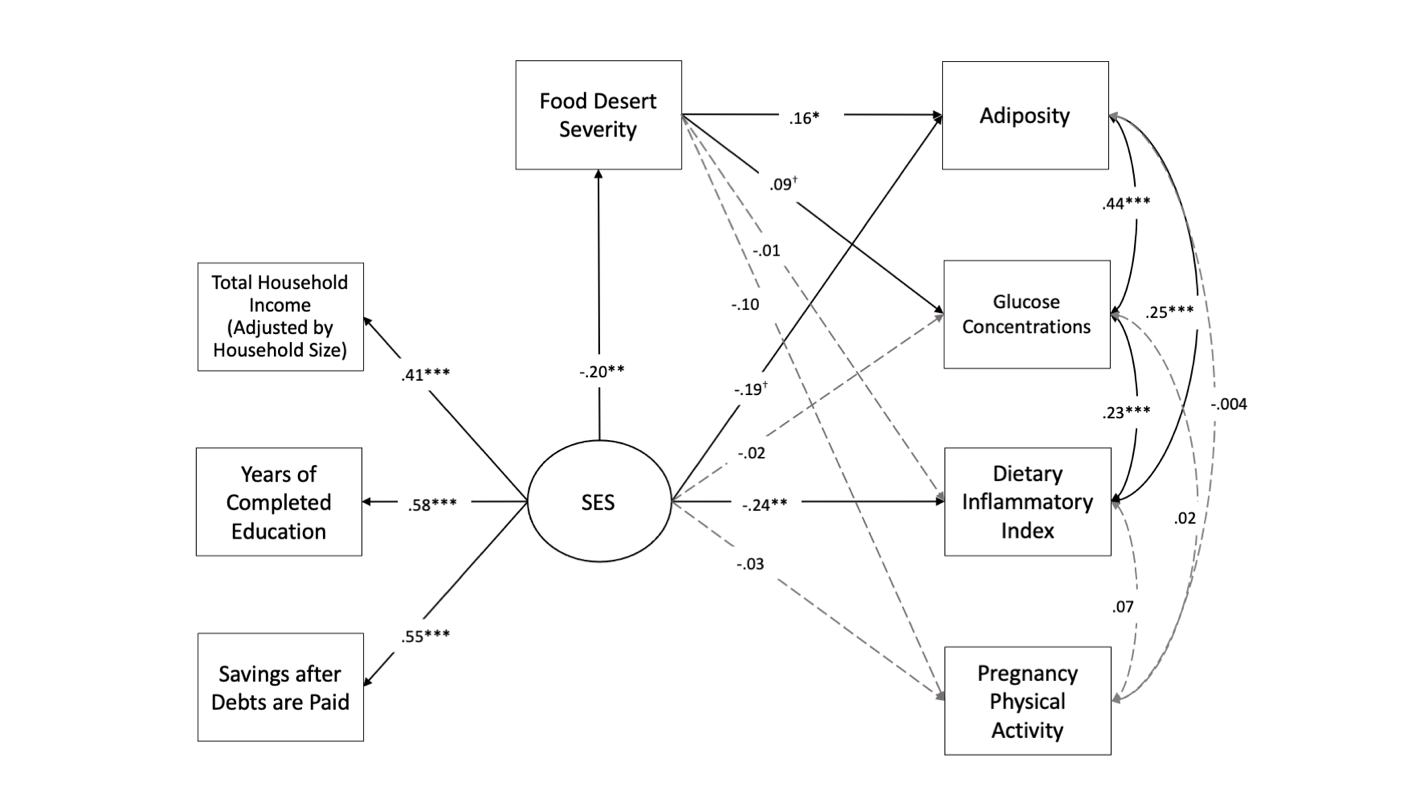


Supplementary Figure S7. *Supplementary Figure S7 Caption.*

Food desert severity scores significantly mediated the relationship between latent SES and adiposity (ß_indirect_= -.03, 95% CI [-.081, -.005]), after adjusting for age of birthing parent, parity status, and racial/ethnic minority status, as well as for weeks’ gestation at GTT, use of alcohol after pregnancy was known, and use of prenatal vitamins. Lower SES was associated with higher food desert severity scores (β = -.20, *p* = .008) and consumption of a more pro-inflammatory diet (β = -.24, *p* = .004). There was a trending effect of lower SES on higher adiposity (β = -.19, *p* = .074). There was a significant association between higher food desert severity scores and higher adiposity (β = .16, *p* = .029) and a trending effect of higher food desert scores and higher glucose concentrations (β = .09, *p* = .088). There was not a significant relationship between physical activity during pregnancy and SES (*p* = .796) or food desert severity (*p* = .133). Model fit statistics: CFI = 0.969; TLI = 0.951; SRMR = .039; RMSEA = .021, 90% CI [.000 .047], *p* = .968*.* ****p* < .001, ***p* < .01, **p* < .05
